# ROBIN: Reference observatory of basins for international hydrological climate change detection

**DOI:** 10.1038/s41597-025-04907-y

**Published:** 2025-04-18

**Authors:** S. Turner, J. Hannaford, L. J. Barker, G. Suman, A. Killeen, R. Armitage, W. Chan, H. Davies, A. Griffin, A. Kumar, H. Dixon, M. T. D. Albuquerque, N. Almeida Ribeiro, C. Alvarez-Garreton, E. Amoussou, B. Arheimer, Y. Asano, T. Berezowski, A. Bodian, H. Boutaghane, R. Capell, H. Dakhlaoui, J. Daňhelka, H. X. Do, C. Ekkawatpanit, E. M. El Khalki, A. K. Fleig, R. Fonseca, J. D. Giraldo-Osorio, A. B. T. Goula, M. Hanel, S. Horton, C. Kan, D. G. Kingston, G. Laaha, R. Laugesen, W. Lopes, S. Mager, M. Rachdane, Y. Markonis, L. Medeiro, G. Midgley, C. Murphy, P. O’Connor, A. I. Pedersen, H. T. Pham, M. Piniewski, B. Renard, M. E. Saidi, P. Schmocker-Fackel, K. Stahl, M. Thyer, M. Toucher, Y. Tramblay, J. Uusikivi, N. Venegas-Cordero, S. Visessri, A. Watson, S. Westra, P. H. Whitfield

**Affiliations:** 1 UK Centre for Ecology & Hydrology, Wallingford, United Kingdom; 2 Irish Climate Analysis and Research Units (ICARUS), Dept. Of Geography, Maynooth University, Maynooth, Ireland; 3 Polytechnic Institute of Castelo Branco, Castelo Branco, Portugal; 4 Institute of Earth Science (IES), University of Évora, Évora, Portugal; 5 Centre for Climate and Resilience Research (CR)2, University of Chile, Santiago, Chile; 6 University of Parakou, Parakou, Benin; 7 Swedish Meteorological and Hydrological Institute, Norrköping, Sweden; 8 University of Tokyo, Tokyo, Japan; 9 Gdansk University of Technology, Gdansk, Poland; 10 Gaston Berger University, Saint-Louis, Senegal; 11 University of Annaba, Annaba, Algeria; 12 LMHE, Ecole Nationale d’Ingénieurs de Tunis, Université Tunis El Manar, Tunis, Tunisia; 13 Ecole Nationale d’Architecture et d’Urbanisme, Université de Carthage, Carthage, Tunisia; 14 Czech Hydrometeorological Institute, Prague, Czechia; 15 Nong Lam University, Ho Chi Minh City, Vietnam; 16 King Mongkut’s University of Technology Thonburi, Bangkok, Thailand; 17 International Water Research Institute, Mohammed VI Polytechnic University, Ben Guerir, Morocco; 18 Norwegian Water Resources and Energy Directorate, Oslo, Norway; 19 Pontificia Universidad Javeriana, Bogotá, Colombia; 20 University of Abobo-Adjamé, Abidjan, Côte d’Ivoire; 21 Czech University of Life Sciences Prague, Prague, Czechia; 22 University of Canterbury, Christchurch, New Zealand; 23 Swiss Federal Office for the Environment, Ittigen, Switzerland; 24 University of Otago, Dunedin, New Zealand; 25 University of Natural Resources and Life Sciences, Vienna (BOKU), Vienna, Austria; 26 Bureau of Meteorology, Canberra, Australia; 27 National Water and Sanitation Agency (ANA), Brasilia, Brazil; 28 Cadi Ayyad University, Marrakech, Morocco; 29 Universidad Politécnica de Madrid, Madrid, Spain; 30 Stellenbosch University, Stellenbosch, South Africa; 31 Da Nang University of Science and Technology, Da Nang, Vietnam; 32 Warsaw University of Life Sciences, Warsaw, Poland; 33 INRAE, RECOVER, Aix Marseille University, Aix-En-Provence, France; 34 University of Freiburg, Freiburg, Germany; 35 University of Adelaide, Adelaide, Australia; 36 South African Environmental Observation Network, Pretoria, South Africa; 37 Research Institute for Development (IRD), Montpellier, France; 38 Finnish Environment Institute (SYKE), Helsinki, Finland; 39 Chulalongkorn University, Bangkok, Thailand; 40 University of Saskatchewan, Saskatchewan, Canada; 41 Present Address: Institute of Earth Science (IES), University of Évora, Évora, Portugal

**Keywords:** Hydrology, Hydrology

## Abstract

Human-induced warming is modifying the water cycle. Adaptation to posed threats requires an understanding of hydrological responses to climate variability. Whilst these can be computationally modelled, observed streamflow data is essential for constraining models, and understanding and quantifying emerging trends in the water cycle. To date, the identification of such trends at the global scale has been hindered by data limitations – in particular, the prevalence of direct human influences on streamflow which can obscure climate-driven variability. By removing these influences, trends in streamflow data can be more confidently attributed to climate variability. Here we describe the Reference Observatory of Basins for INternational hydrological climate change detection (ROBIN) – the first iteration of a global network of streamflow data from national reference hydrological networks (RHNs) – comprised of catchments which are near-natural or have limited human influences. This collaboration has established a freely available global RHN dataset of over 3,000 catchments and code libraries, which can be used to underpin new science endeavours and advance change detection studies to support international climate policy and adaptation.

## Background & Summary

Future climate projections suggest hydrological extremes (floods and droughts) will become more frequent and severe^1–8^ – further intensifying the impacts they have on livelihoods, infrastructure, and economies. To adapt to future changes in these extremes, we need better projections of future flood and drought occurrence. Hydrological models are used in the production of such scenarios, but they can be very complex and highly uncertain^9^. To better understand and constrain these model-based projections, we need to quantify emerging trends in the water cycle based on observed data. This requires long records of past hydrological observations. Streamflows are especially useful because they integrate climate processes over large areas covered by drainage basins.

Across the world, there have been many studies of long-term trends in streamflow^10–12^. Despite this past research, our confidence in observed trends remains low – even in the state-of-the-art Intergovernmental Panel on Climate Change (IPCC) reports, which have typically been cautious in their conclusions regarding long-term changes in floods or droughts. One reason is that most rivers are heavily modified by human disturbances^13^ (e.g., dams, large removals of water for irrigation, domestic or industrial consumption). These disturbances can obscure the ‘signal’ of climate change – that is, trends in many rivers may bear little relation to global warming and may in fact be opposing the climate trend, due to human modifications such as dam construction^14^.

To overcome these barriers of the confounding impact of anthropogenic disturbances, many countries have established ‘Reference Hydrometric Networks’ (RHNs)^15–20^, consisting of catchments that are relatively undisturbed (to a greater or lesser degree) and gauged by stations with high quality data. The concept of RHNs, their history and evolution are described previously^15–21^, and a wealth of studies^10^ have been published describing trends and variability at such datasets at the national scale.

Another key barrier to global scale analysis of streamflow variability is the lack of coherence between national- or regional-scale streamflow datasets, and the lack of consistent methods used to analyse variability. High-quality, global datasets are essential, and also exist in the literature. Examples include the Global Runoff Data Centre (https://www.bafg.de/GRDC), which provides river discharge estimates at 10,000+ sites, and the Global Streamflow Indices and Metadata Archive (GSIM) which provides metadata and indices derived from more than 35,000 daily streamflow time series. Efforts have also been made to quality check more than 21,000 timeseries of river-flow worldwide merging multiple open-access sources^22^. Where ROBIN differs is that the focus has been on building the network focussing only on natural and near-natural basins with national experts embedded to enable the separation of the climate signal from anthropogenic influences. There have been previous efforts to bring together RHNs at the supra-national scale and analyse climate variability using them (e.g., European^23^ and Transatlantic^24^ scale studies). Globally, open repositories are used in hydrological climate-impact modelling for e.g. calibration^25^ and sensitivity analysis^26^. However, this is the first attempt to compile reference streamflow data for global climate assessments based purely on observations. The ambition is to expand the ROBIN network over time.

**Fig. 1:**
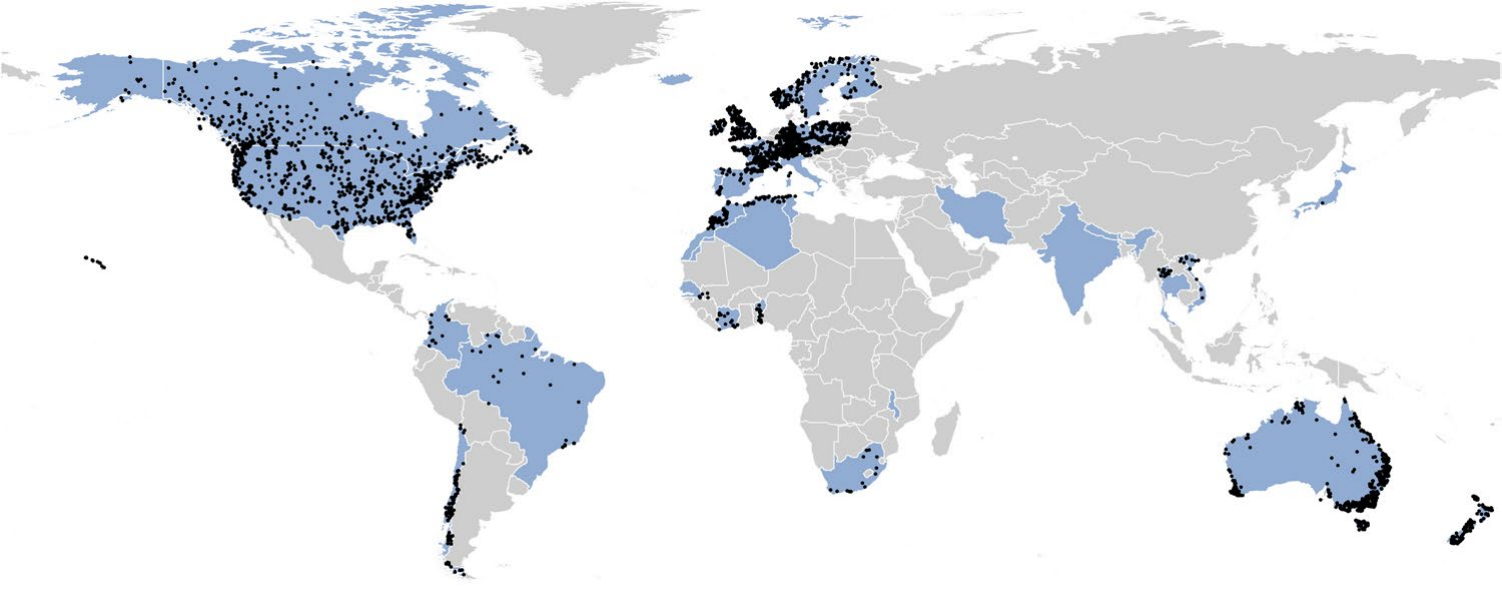
Spatial distribution of countries participating in the ROBIN Network in March 2025 (blue shading). Black dots are stations included in the first iteration of the ROBIN dataset.

To advance reliable global assessments of streamflow variability such as those sought by the IPCC, there is a need for much wider RHNs or catchments with RHN-like status. There is also a need for an integrated and consistent approach to allow robust international and ultimately global comparisons. To this end, in 2021 the **R**eference **O**bservatory of **B**asins for **In**ternational hydrological climate change detection (ROBIN) initiative^27^ established a new long-term collaboration of international experts to develop global capacity for establishing and sustaining RHNs, whilst sharing best practice and skills to create the underpinnings for a global RHN through common standards, protocols, indicators and data infrastructure. The ROBIN initiative aims to develop a widely available global RHN dataset, as well as code libraries which can be used to underpin new science and advance change detection studies to support international climate policy and adaptation, including future IPCC reports. As well as being a network of hydrometric data, ROBIN is also a network of people. As of March 2025, 67 experts are engaged with the establishment, processing and analysis of data from RHN-like catchments. This paper describes the first version of the ROBIN dataset of relatively undisturbed, high quality streamflow observations. The first iteration of the ROBIN dataset consists of daily streamflow data from 3,060 gauging stations from 30 countries (shown in Fig. 1).

Inevitably with such an ambitious and far-reaching data initiative, the ROBIN dataset currently only includes a relatively limited sample of countries and there are clearly many gaps in this first version (although as described below, there is encouraging coverage and representativeness of the global land surface). Future versions of the ROBIN Network will aim squarely at extending this concept and coverage to as many countries and unsampled regions as possible.

This paper describes the ROBIN dataset and does not describe the analysis of the data. The dataset is designed to support a wide range of analyses of trends and variability, though a host of other applications can be conceived, as we will revisit in the final section of this paper. Analysis of global trends in low flows and the characteristics of droughts in this dataset is currently underway by the ROBIN team, however it is hoped the dataset can be taken forward by many researchers across the global hydrology community (and many other disciplines and sectors besides) to conduct their own analyses. We also plan to provide continual updates to the dataset with the inclusion of more countries and stations giving better coverage across the globe.

## Methods

The ROBIN Network aims to develop a truly global, long-lasting RHN network. While RHNs are well established in many countries, in many others, efforts to establish RHNs are in early stages, or have not commenced. This first iteration of the ROBIN network was therefore conceived as a way of bringing together experts from a wide range of countries and climates with established RHNs, nascent RHN or RHN-like catchments – and, simply, to bring together researchers and practitioners from countries with no RHN but a willingness to participate in establishing an RHN or at the very least, RHN-like catchments suitable for a global scale dataset.

Founding members of the ROBIN Network have previously collaborated in efforts to establish networks of RHN catchments, or at least RHN-like catchments, across international boundaries^23,24^. The collaborators in these initiatives became the core team of ROBIN from its inception in 2021. The ROBIN Network of countries and experts was then grown through a campaign of establishing contacts in other countries via personal connections, and through wide promotion of ROBIN in the international community e.g. AGU, EGU, IAHS, online webinars, UNESCO-IHP FRIEND.

In past international ventures to pool RHN datasets, it has often proved difficult to develop a suitable set of criteria for inclusion of stations – not least because there can be very different definitions of what constitutes a ‘natural’ catchment and ‘good quality data’ between (and sometimes within) countries. In some parts of the world RHNs can be based on truly ‘pristine’ unaltered catchments, whereas in a majority of localities, some degree of human disturbance must be tolerated. Moreover, there is always a trade-off between ensuring an acceptably low level of disturbance or good quality data on the one hand, and having RHN criteria that are too exacting, which results in a limited number of stations and a reduction of coverage and representativeness of the network. Within ROBIN we aimed to balance the need for near-natural catchments against network density, and therefore defined an inclusive two-level approach to the criteria for gauging stations to be included within the ROBIN Network.

These two levels were assessed by in-country experts and are intended to give a more flexible approach to balance the requirements of robust data analysis with good coverage of global geographies and hydrological regimes. In the case of Level 1 criteria, they built directly upon the criteria used a similar previous study^28^. The ROBIN consortium held multiple workshops in 2022 to develop these initial criteria, sharing experiences of the realities of defining RHN or RHN-like catchments, and worked collaboratively to agree on the final criteria for stations to be included in the dataset. The results of these discussions was the network criteria shown in Table 1. Notes from the workshops are available on the ROBIN website (https://www.ceh.ac.uk/our-science/projects/robin).

**Table 1:** Summary of ROBIN Network Criteria.

Level 1 Network	Level 2 Network
Largely free from human disturbances such as urbanisation (≤10% of the catchment), river engineering and water abstractions. Modest net impact of all influences on low flows and high flows and any impacts stable over time. No known major changes in land use likely to impact streamflow regime.	Fairly free from human disturbances such as urbanisation (≤20% of the catchment), river engineering and water abstractions. Modest net impact of all influences on monthly and annual flows and any impacts stable over time.
Very high-quality daily mean river flow data capable of reliably representing high and low flows. Appropriate metadata.	High to fair quality daily mean river flow data capable of reliably representing monthly average flow conditions with appropriate metadata.
Record length of at least 40 years	Record length of at least 20 years
No data gaps longer than three years.	

The intention of the two-level approach was also to guide users towards appropriate usage of the data, for example:

- Data from Level 1 gauging stations could be focussed towards analysis of extreme flows (both high and low) where the highest quality and most complete data from ‘pristine’ catchments (or as close to this as possible) will be required.
- Data from Level 2 gauging stations could be used for analysis of less sensitive hydrological variables such as monthly, seasonal or annual mean flows and water balances.

The criteria are in many ways qualitative in nature, which allows for a degree of flexibility in station inclusion, and we accept that compromise may be required to ensure there is a geographically representative network for the globe. The assessment levels stated in the criteria (very high, high, fair) are relative to the country and not dataset-wide (i.e. that “very high quality” in country X may carry different implications than it does in country Y). While this qualitative nature was an advantage for inclusivity, there are inevitably still (arguably insurmountable) differences in interpretation which must be borne in mind. To this end, the community nature of ROBIN was fundamental in ensuring consistency as far as possible. As well as the aforementioned workshops, a range of other peer-to-peer mechanisms were implemented, including regular Q&A clinics, a centralised email address for the coordinating team to field enquiries and to disseminate guidance on the RHN criteria. Language barriers were a challenge, and the ROBIN Network attempted to overcome such hurdles through having partners who could represent wider domains (e.g., native French speakers assisting in disseminating guidance in Francophone countries in Africa).

Overall, the local knowledge of ROBIN partners was fundamental to ensuring the network is as representative as possible and the stations included were appropriate. Notwithstanding some inevitable differences in interpretation, the key strength of ROBIN is the inclusion of at least one (and often several) expert(s) with local-scale knowledge. As well as enabling the designation of ROBIN status, this also ensures the local expert can be the nexus between the international objectives of ROBIN and the unique local-scale hydrology, governance and data management settings, regional and national imperatives and so on. This is not a unique property of ROBIN but contrasts with some other international data sharing initiatives in the geosciences which aim to facilitate data transfers, but do not incorporate and build on local-scale knowledge.

Whilst the first iteration of ROBIN aimed to provide a truly accessible global dataset, there are inevitably still challenges in the sharing of hydrometric data at an international level^29,30^. In many jurisdictions, it was simply not possible to share data outside of a given country or region due to legal or commercial constraints. Hence, to overcome these challenges, ROBIN has adopted a tiered approach to data sharing, whereby participant countries can share data at a suitable level of accessibility to meet national/regional constraints. Where no data could be shared, a Code Library (https://github.com/NERC-CEH/ROBIN_pipeline) was developed to allow extraction of indicators that can be analysed centrally. The different tiers of data access are:

- ROBIN Public Dataset (2,386 stations)^31^ – where daily river flow data are freely available and incorporated into the open DOI dataset.
- ROBIN Full Dataset (3,060 stations) – where only the locations and metadata of the catchments are shared centrally in the DOI dataset, but the river flow data (or relevant indicators) are made available to other members of the ROBIN Network.

Access to the ROBIN Full Dataset is limited to those in the ROBIN Network, as a consequence of agreements entered with national data providers. For the moment, ROBIN Network membership must remain limited to experts who have provided data or substantial local knowledge to the network.

The ROBIN dataset also includes a range of metadata variables, as described in the following section. ROBIN partners provided basic metadata, such as site ID and name, location and catchment area. Where possible, catchment boundary shapefiles were also provided to assist with the derivation of metadata from global datasets and these were provided by 9 out of 30 countries. For the remaining 21 countries, catchment boundary shapefiles were derived using the given location of the gauge and the HydroSHEDS global ~450 m (15 arc second) grids^32^. Boundaries derived from HydroSHEDS were accepted where the computed area had a difference of less than 15% compared to the catchment area submitted by the host country. Where the difference in area was more than this, the surrounding grid cells were checked to see if there was a closer match available, and if so that location was taken forward. In a few instances (less than 100 catchments) the catchment outlet was moved by two or three squares from the original location given by the host country. Not all catchments could have catchment boundaries derived due to issues with given locations not being close to a river and very small catchments that did not resolve well on the ~450 m grid used.

Once catchment boundaries had been derived for as many ROBIN stations as possible (2,971 out of 3,060), tools from the CARAVAN programme^33^ were used to extract catchment attributes for all stations to enhance the metadata available within ROBIN to ensure suitability for a range of hydrological applications. The catchment attributes from CARAVAN relate to the climate, soils and geology, land cover, hydrology, physiography, and anthropology. Where suitable catchment boundaries had been submitted, these were used in the metadata extraction process, otherwise the catchment boundaries generated from HydroSHEDS were used. As an initial suitability check, the CARAVAN derived catchment area was compared with the submitted catchment area for a handful of countries and the differences found to be minimal enough (<10%) to proceed. The CARAVAN-provided code (available from the GitHub repository: https://github.com/kratzert/Caravan) was then run for as many stations as possible; this extracted static catchment attributes, derived from HydroATLAS, based on the uploaded catchment shapefiles. All default attributes in the CARAVAN notebooks were extracted for the ROBIN catchments, rather than selecting only certain attributes or attribute types, in order to keep the possible uses of the ROBIN dataset as wide as possible – a full list of these attributes is available from CARAVAN^33^ and in the Supporting Documentation provided alongside the ROBIN dataset. CARAVAN data are provided for as many stations as possible in the ROBIN Dataset (2,865 out of 3,060). The 6% of stations without CARAVAN data are due to not being able to produce catchment boundaries at some sites (5%), and other unspecified errors faced when trying to extract the metadata, we believe due to the size of catchments (too large or too small; 1%). It is also possible within CARAVAN to extract time series of meteorological forcing data from ERA5-Land, although it was decided that this was beyond the metadata requirements for the ROBIN dataset at this stage – however, this is for a possible future ROBIN extension.

Within the first version of the ROBIN Full Dataset there are 3,060 stations providing daily streamflow data (m^3^/s) and associated metadata records; a subset of these (2,386) are available in the ROBIN Public Dataset^31^ where the daily streamflow data (m^3^/s) are openly available. The number of stations with streamflow data per country in the Full and Public datasets is shown in Table 2.

**Table 2:** Number of stations within the ROBIN Full Dataset (3,060) and ROBIN Public Dataset (2,386) per country.

Country	ROBIN Full Dataset	ROBIN Public Dataset^31^
Algeria	32	—
Australia	452	452
Austria	45	45
Benin	19	19
Brazil	24	24
Canada	315	315
Chile	104	104
Colombia	14	14
Czechia	16	16
Côte d’Ivoire	14	—
Finland	26	26
France	207	207
Germany	336	—
Ireland	23	23
Japan*	1	—
Morocco	60	—
New Zealand	111	83
Norway	116	116
Poland	136	—
Portugal	16	16
Senegal	5	—
South Africa	14	—
Spain	16	—
Sweden	15	15
Switzerland	50	50
Thailand	15	—
Tunisia	5	—
United Kingdom	146	146
United States of America	715	715
Vietnam*	12	—
Total	3060	2386

*Indicates countries which could not share streamflow data at all and are solely metadata records.

## Data Records

The Public ROBIN dataset^31^ is available from the Environmental Information Data Centre under the terms of the Open Government Licence - 10.5285/3b077711-f183-42f1-bac6-c892922c81f4. Alongside the streamflow data, basic metadata is provided from the measuring organisations including station ID, name, locational information, and catchment area. Stations were then given a ROBIN ID comprising of the UN/LOCODE Code List ISO 3166-1-alpha-2 code element, plus a consecutive digit in the format e.g., GB00001. The metadata derived from the CARAVAN products is also included here. The streamflow data for each station is presented in a CSV file which consists of a header row with the column names robin_id, date (dd/mm/yyyy), flow (m^3^/s). The data are then presented underneath the header row. Missing data are shown as NULL.

**Fig. 2:**
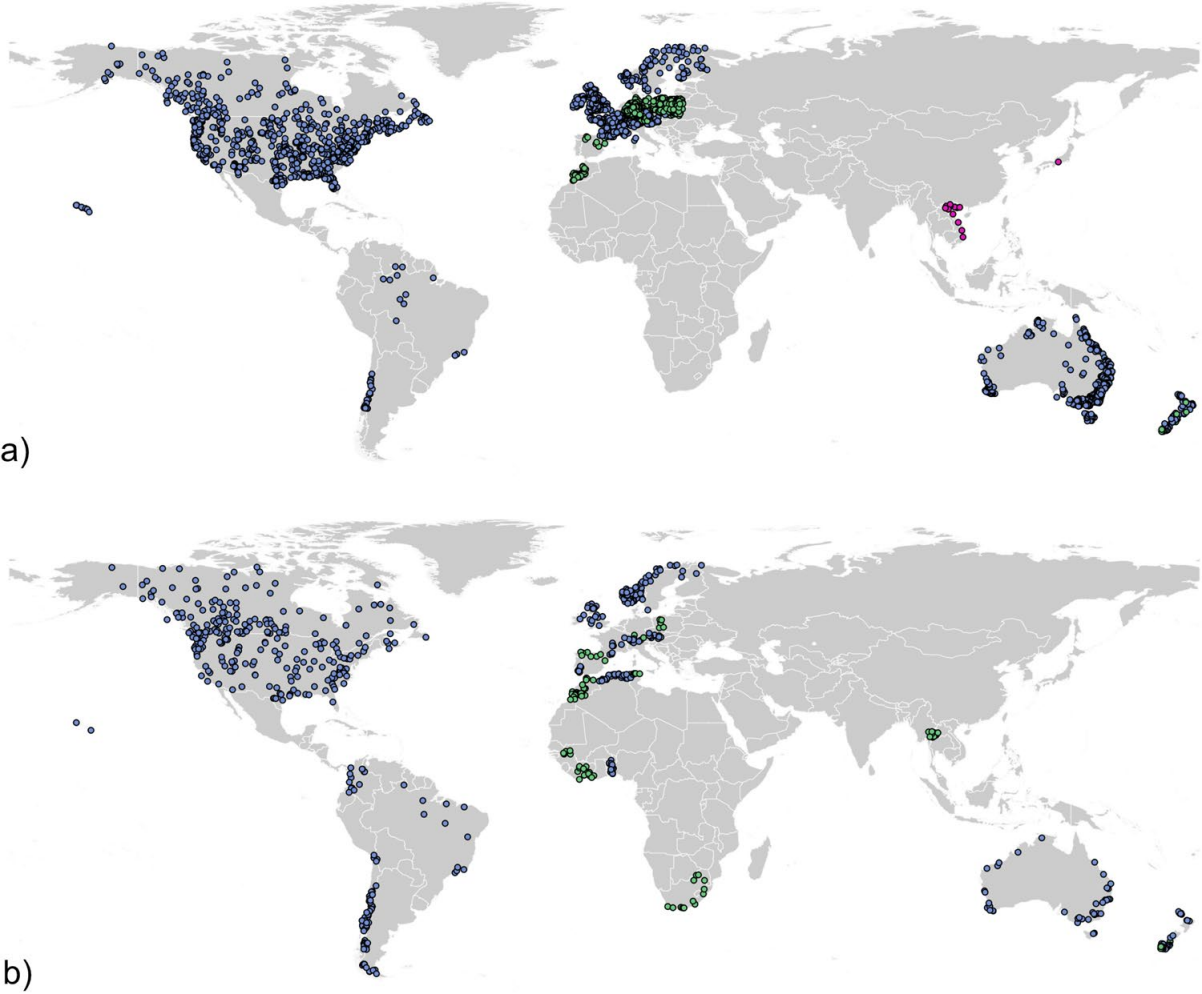
Spatial distribution of stations in (**a**) Level 1 (2,313) and (**b**) Level 2 (747). Blue dots show stations in the Public dataset, green dots show stations only available in the Full dataset and pink dots are metadata only records.

Stations were assigned to the Level 1 or Level 2 groups by the local specialists (and where stations were in a previous related study^28^ these were assigned Level 1), and then all stations were run through the quantitative record length and completeness criteria to get the final Level 1 and 2 listings (Fig. 2).

## Technical Validation

The ROBIN Full Dataset contains daily observed streamflow data from 3,060 stations across the globe (with 2,386 stations being available in the ROBIN Public Dataset^31^). Following submission of data and metadata to the ROBIN Network from each country, a quality control process was conducted centrally. As the ROBIN project includes many of the same collaborators as a previous related study^28^, it was deemed that the stringent quality control undertaken through the earlier project would be sufficient for ROBIN stations, and the close collaboration allowed the team to ensure the quality control was carried out in a similar manner. Hence 1,751 stations passed from the previous study^28^ into ROBIN without further QC.

**Fig. 3:**
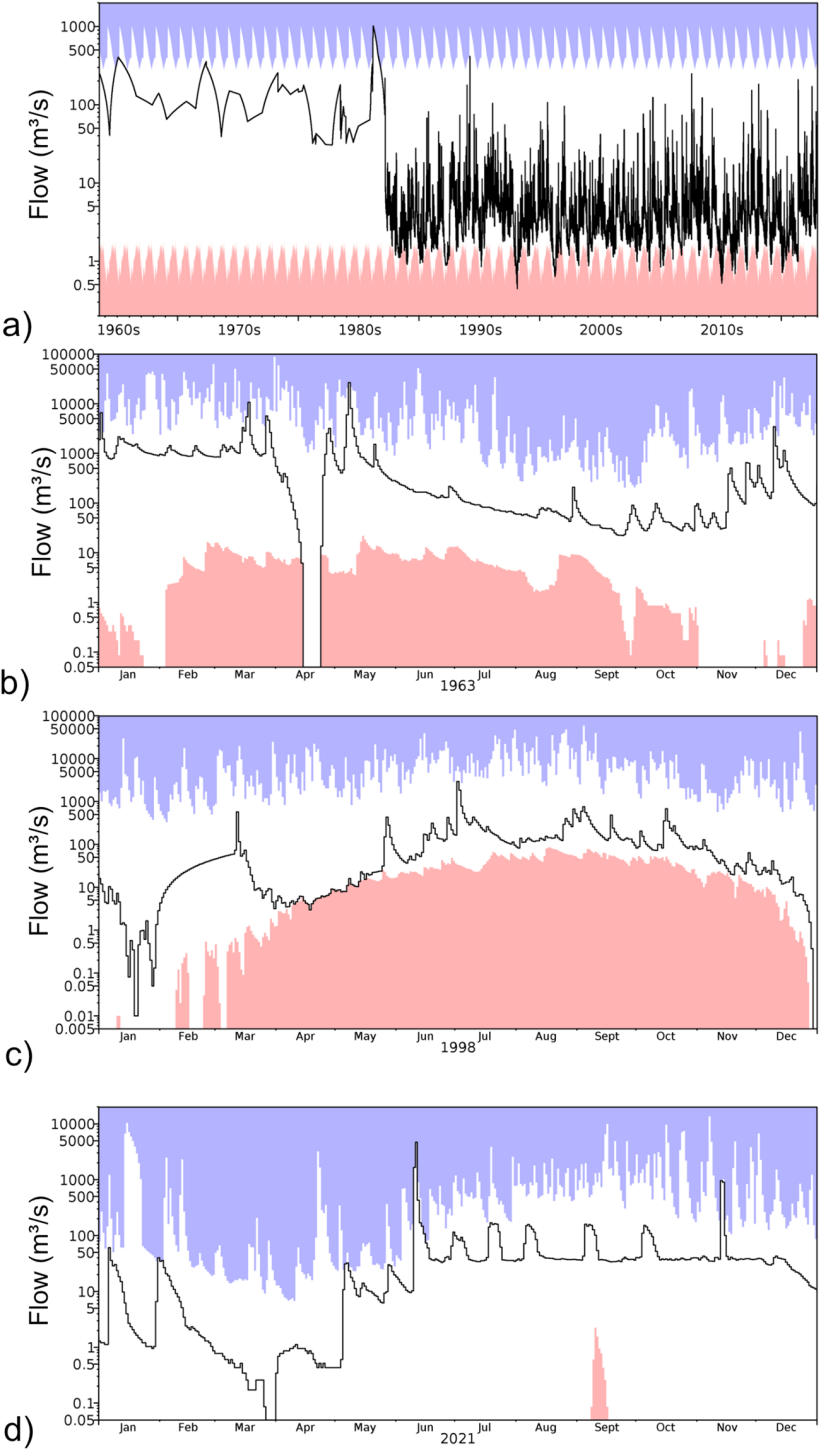
Examples of issues found during the QC period: (**a**) step change halfway through a multi-year record (in addition to a different interpolation method during the earlier period) (**b**) a sudden drop in flows, just for a few days from a high flow; (**c**) linear infill over a long period of time looks out of place (between Feb and Mar 1998) – perhaps infilling erroneous or missing data following freezing during winter; (**d**) potentially unnatural regime present in flows. Envelopes show highest (blue) and lowest (pink) flows on record for each day of the year. Note log scale of y-axis on all hydrographs to emphasise low flows.

The 1,296 stations that were not part of the previous related study^28^ underwent a comparable manual quality control process to assess the quality and suitability for inclusion in the ROBIN dataset. The quality control process was based on the same principles as applied in the previous study and was carried out by a team of three hydrologists who are part of the quality control team for the UK National River Flow Archive, using the QC software used in practice by the national hydrometric data centre^34^. Plots of daily streamflow records were visually screened for change points, visually anomalous conditions (indicating methodology changes or infilled data gaps) and obvious errors. Stations were removed if they showed signs of not having a sufficiently ‘near-natural’ regime, but edits were made over datasets to remove obviously erroneous data periods – this was especially useful as it allowed the bulk of ‘good’ data to remain in the ROBIN dataset. Some examples of the types of data that were removed from the dataset can be found in Fig. 3. Acknowledging that having three separate people undertaking the quality control could lead to subjectivity issues, any questionable cases were discussed in a follow up session between all three members of the QC team where a consensus decision was made. The QC process was carried out to screen the dataset of any major issues. We are aware that some issues may still be present in the ROBIN dataset and would encourage any concerns to be sent to the authors so they can be addressed in future iterations of the dataset. Following the quality control stage, the cleaned timeseries and metadata were assembled into an ORACLE database.

The designation of RHN-like criteria and rigorous quality control undertaken by the host countries and ROBIN team ensures the dataset is as high quality as possible and is believed to be largely free of gross errors. However, measuring river flows inherently comes with its challenges and associated uncertainties, particularly in the extreme flow ranges^35^. It is also worth noting that recent studies have pointed to the pitfalls of quality control using expert judgment, with widely varying outcomes between observers^36^. Visual appraisal by trained hydrologists (i.e. a small team in the case of ROBIN) is preferable to automated checks – although future iterations may benefit from a hybrid of human judgment and emerging machine-learning based methods.

It is not possible to rule out some degree of error, nor possible to completely rule out anthropogenic influences on streamflow, despite the criteria employed in designating the dataset. As noted in the introduction and methodology, the criteria around human influences are, necessarily, somewhat subjective and open to interpretation, and there are widely varying views of what constitutes ‘undisturbed’. For some parts of the world, the information available to ‘outsiders’ to make such judgments is sometimes lacking, and the extent of impacts is simply unknown or potentially unknowable given, for example, a lack of documentation of water withdrawals. Finally, our criteria focuses on large-scale ‘direct’ modifications like dams or substantial withdrawals, and so we cannot rule out the role of catchment-scale land use/land management changes, other than via our criteria intending to filter out catchments with large degrees of urbanisation or *known* large-scale changes. In reality, there are comparatively few truly undisturbed areas, and even remote rural locations may have experienced some changes over the ROBIN study period – for example, some studies^37–43^ have highlighted the potential role of gradual changes.

Despite these constraints, we believe ROBIN represents an important advance in accessible, global-scale high quality streamflow datasets with a strong commitment to ensuring anthropogenic disturbances are as minimal as possible.

**Fig. 4:**
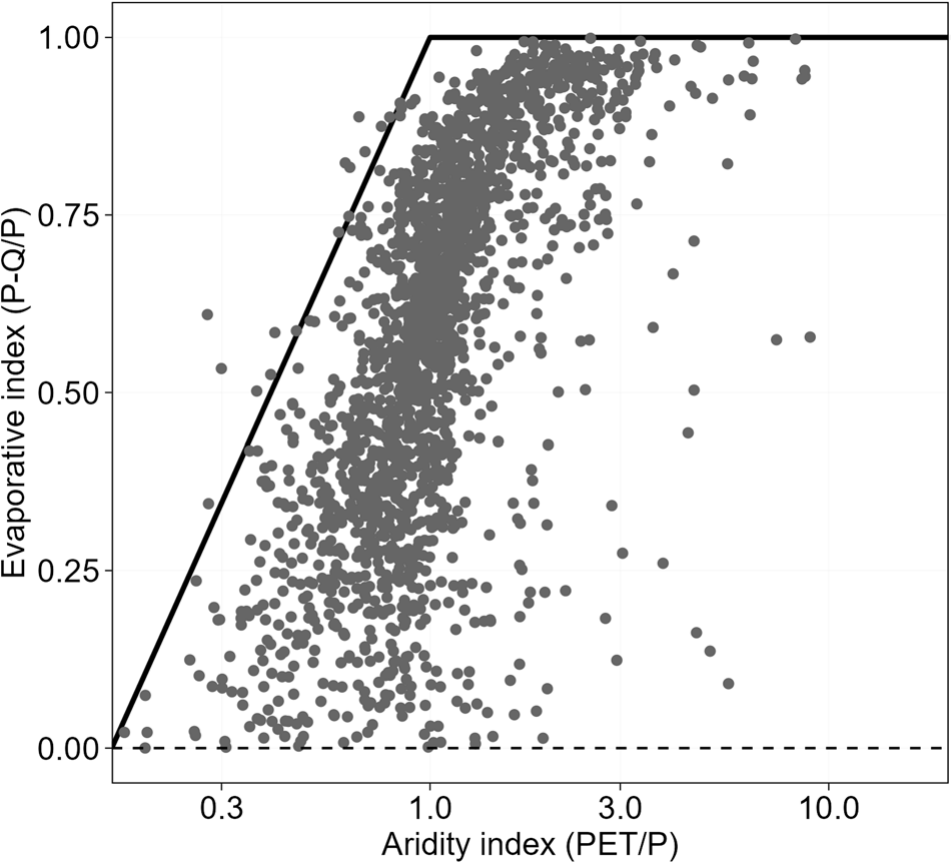
Budyko space locations of the 3,060 ROBIN stations over the 1981–2010 period, showing that 83% of stations have a sensible water balance calculation.

In addition to the manual quality control checks, a more quantitative evaluation of the ROBIN dataset was undertaken to give further confidence that flows are reasonable for near-natural basins and free from any major errors. Firstly, the data were plotted according to the Budyko framework^44^ to assess whether water balance calculation appeared sensible (Fig. 4). Using flow data from the ROBIN Full dataset and catchment-averaged rainfall from ERA5-Land provided by CARAVAN over the 1981–2010 period, each station’s mean evapotranspiration (expressed through the evaporative index) is plotted against the aridity index (calculated using catchment averaged PET from ERA5-Land). The Budyko framework sheds light on the partitioning of rainfall to streamflow and its sensitivity to climatic changes or catchment-specific influences (e.g. land use change). This simple water balance evaluation shows that the majority of stations (83%) appear to fall within expected locations on the Budyko space over the 1981–2010 period and the station data appears free from any major errors. The trajectories of individual stations across the Budyko space over time merits further investigation in future work. There are a number of stations with a negative evaporative index (i.e. Q > P) but these can be attributed to uncertainties in the global rainfall product^45^ used (ERA5-Land), anomalies in provided catchment area, or more specific issues such as stations in karstic environments where a ‘traditional’ water balance calculation is more difficult.

**Fig. 5:**
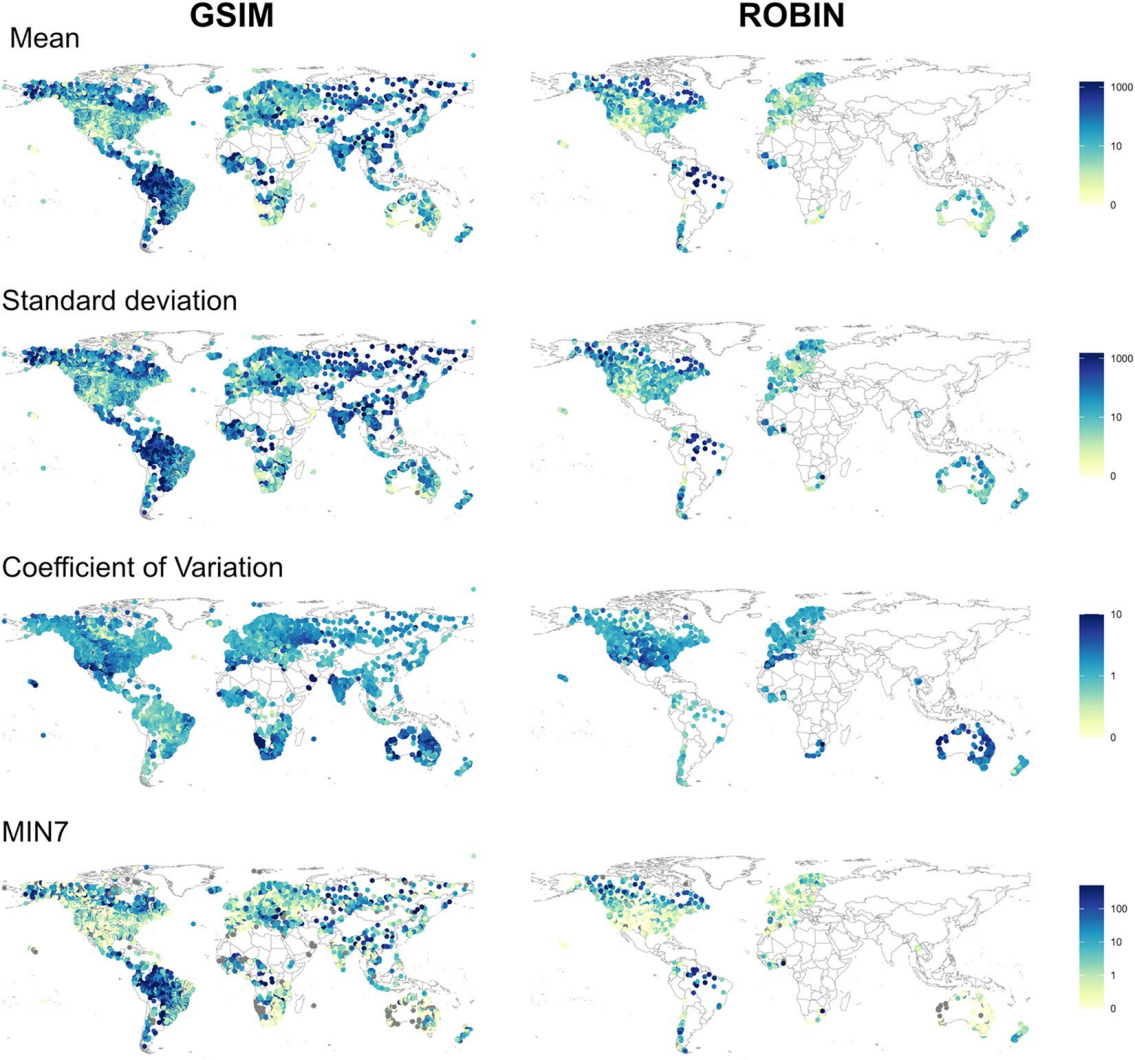
Comparison of GSIM and ROBIN stations for four streamflow indicators (from top to bottom; mean flow, standard deviation, coefficient of variation and 7-day minima flows. Streamflow metrics for ROBIN stations are calculated using the full record length of each station.

Secondly, streamflow indicators from ROBIN stations were compared to another large-sample hydrology dataset - GSIM^46^ (Fig. 5). Once again, there are many similarities between ROBIN and GSIM which is encouraging to give confidence to the ROBIN dataset. Where there are deviations between the two, this could be attributed to the fact that ROBIN only includes natural or near-natural sites, whereas GSIM includes, natural and influenced sites. GSIM sites therefore cannot be directly compared to the ROBIN stations. Nevertheless, the purpose of this comparison is to identify any clear anomalous outliers in the respective hydrological signatures rather than to ensure similarity between ROBIN and other large sample hydrology datasets. The comparison with GSIM suggests that streamflow indicators for ROBIN stations are within the range expected from the different hydroclimate regions. For example, stations in the Amazon basin exhibit greater mean streamflow values and stations across Australia have a highly variable hydrological regime with a high coefficient of variation.

**Fig. 6:**
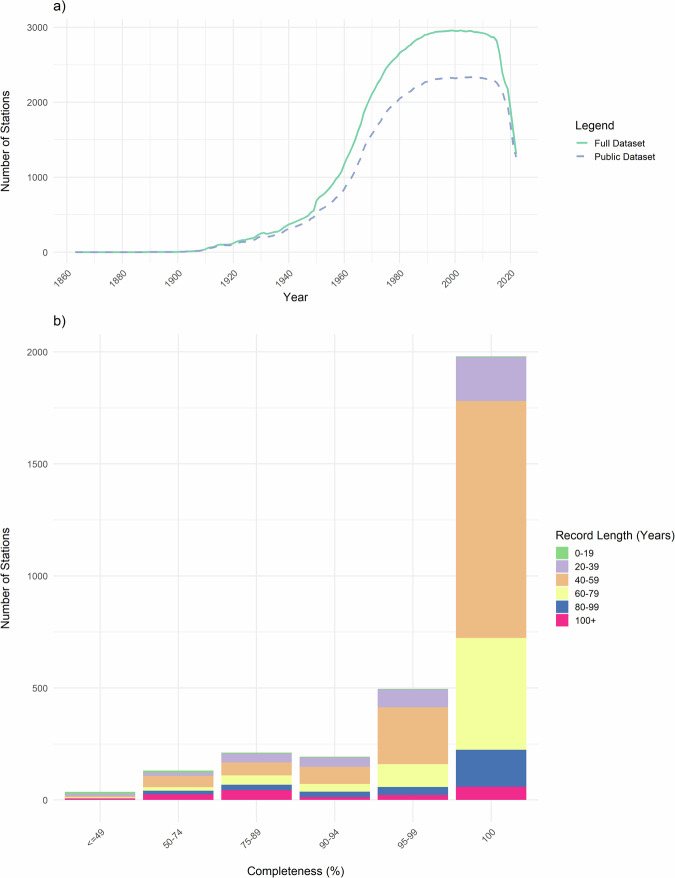
Summary statistics show (**a**) number of active ROBIN stations through time; Green = ROBIN Full Dataset, Dashed Blue = ROBIN Public Dataset^31^, (**b**) completeness and record length of records in the ROBIN Full Dataset.

The dataset summary statistics (Fig. 6) show the number of active ROBIN stations in the ROBIN Full Dataset over time (green trace), and the number of active ROBIN stations in the ROBIN Public Dataset^31^ (dashed blue trace) (6a). The vast majority of the stations (88%) have records which are ≥90% complete, whilst the median record length is 50–55 years. It is encouraging that a majority of the gauges within ROBIN (73%) have a completeness of 95% and a record length of 40 years or more (6b). These attributes are crucial for ensuring that the ROBIN dataset is fit for purpose for analyses of long-term trends in streamflows.

Spatially, the dataset covers all the major Hydrobelts^47^ (i.e., delineated homogenous hydrological regions across the continents) as shown in Table 3. The majority of stations are in the Northern Mid Latitude which reflects the fact that these areas within North America and Europe are some of the densest hydrometric networks in the world. There is, however, representation from all Hydrobelts. The least represented Hydrobelt is the Equatorial region (29 sites). Temporally, the average record length for stations in all Hydrobelts is around 40 years or more, with many regions having data records starting in the early 20^th^ century or earlier. With future iterations of the ROBIN Network, these summary statistics will be used to influence areas to target to increase representativeness.

**Table 3:** Summary information on the number of stations, record lengths, size of catchments by Hydrobelt region.

Code	Hydrobelt name	No. of Stations	Earliest Date	Latest Date	Max. Record Length	Min. Record Length	Mean Record Length	Total Area (KM^2^)	Small Catchments (<100 KM^2^)	Medium Catchments (>=100 to <1000 KM^2^)	Large Catchments (>=1000 KM^2^)
BOR	Boreal	270	01/01/1863	31/12/2022	158	20	59	2258807	40	93	137
EQT	Equatorial	29	01/01/1955	26/05/2022	65	10	39	2515682	2	6	21
NDR	Northern Dry	162	01/01/1913	31/12/2022	110	14	50	187662	32	89	41
NML	Northern Mid Latitude	1781	01/01/1892	31/12/2022	130	15	63	1095576	597	931	253
NST	Northern Sub Tropical	115	01/10/1907	31/12/2022	115	11	52	624250	25	42	48
SDR	Southern Dry	101	01/09/1918	28/02/2022	102	25	47	676004	10	54	37
SML	Southern Mid Latitude	534	01/03/1929	31/12/2022	91	21	51	488106	81	348	105
SST	Southern Sub Tropical	55	01/10/1934	29/09/2022	84	35	55	520768	8	24	23

## Usage Notes

We believe ROBIN represents a significant advance in making global-scale near-natural streamflow data accessible. There are numerous national-scale RHNs available, but this is the first effort to establish a readily available international RHN-like dataset, building on previous efforts to provide European-scale RHN-like stations^23^.

The primary focus for ROBIN is for large-scale trend detection and attribution and quantification of long-term hydrological variability, following the lead established in European^48^, transatlantic^24^ and wider^49^ efforts. We envisage that ROBIN will be the foundation of a new wave of consistent global-scale analyses of hydrological change.

While ROBIN was motivated by quantifying hydroclimate variability, there are a whole host of other potential uses for such a near-natural, high-quality dataset. Near-natural records are obviously vital for meeting our established goal of quantifying climate driven changes, but there are many other hydrological or ecological applications where near-natural conditions are sought. Obvious examples in the hydrological sphere include: finding links between river flows and large-scale climate drivers and teleconnections; providing observational benchmarks for hydrological model evaluation; developing statistical regionalisation techniques; and providing baseline conditions for future climate risk estimates.

The recent past has seen an upsurge in efforts to quantify the impact of anthropogenic disturbances on river flows, notably through the IAHS ‘Panta Rhei’ decade^50^. For such applications to quantify anthropogenic disturbances (dams, water withdrawals, etc), there is a need to establish a natural baseline from which to quantify departures to demonstrate the scale of human impacts. To this end, naturalised data can be modelled with uncertainties^51^ while RHNs provide baseline data for comparisons against nearby or analogue catchments. Similarly, the hydroecology community has long employed natural reference conditions, to quantify streamflow alterations due to anthropogenic disturbances^52^. Naturalised data are often difficult to access or unavailable^30^, so near-natural datasets provide one way of establishing baseline conditions via data transfer or regionalisation^53^.

All stations within the ROBIN Full Dataset have been designated as reference, or reference-like stations and therefore should be appropriate for when natural or near-natural basins are required for analysis. The level system (Level 1 and 2) was designed in a way that the Level 1 stations, which have been through a more stringent selection process, can be more confidently used when analysing extreme flows for example, where the highest quality and most complete records would be required, whereas, in order to balance inclusiveness of sites, regions and countries, Level 2 stations could be used to analyse less sensitive hydrological variables such as monthly, seasonal or annual mean flow, or where record-length is a less important criteria. As previously noted, the two-tier approach was employed to help maximise representativeness of the network. There is still obvious under sampling of the network, of which we hope to improve in future iterations of the dataset. There is also a more profound under sampling of river gauge networks more generally^54^. River gauges are often placed on high Strahler stream order reaches which are perennial. Gauges on reaches with high proportions of natural land, and therefore smaller human populations are often under-represented. Whilst this may be a barrier to ROBIN, there is also a benefit of linking model, observations, and earth observation assessments in future.

Our vision for ROBIN is that of a growing network within the global hydrological community, in which users can access and, in turn, develop the dataset and analysis. Whether that is existing members conducting new and novel analyses with the data, or new members joining the Network to add to and expand the spatial and temporal footprint of the dataset.

## Data Availability

The observed streamflow data are made available in the EIDC dataset (10.5285/3b077711-f183-42f1-bac6-c892922c81f4. We have made our source codes publicly available to analyse the ROBIN dataset. The codes are written in R, and can be used to format the data, check for Level 1 / Level 2 status based on completeness, run a selection of summary statistics on the dataset (Monthly thresholds, POT series, 30-day/10-day/7-day rolling totals, Annual/monthly/quarterly quantiles/mean, Annual 1-day/10-day/30-day maxima, Annual 7-day/30-day minima), run statistical trend and breakpoint tests (Pettitt breakpoint test, Seasonal Mann-Kendall, Rolling Mann-Kendall-Snyers Indicators). These can be accessed through the ROBIN GitHub directory (https://github.com/NERC-CEH/ROBIN_pipeline).
