# Author Correction: ROBIN: Reference observatory of basins for international hydrological climate change detection

**DOI:** 10.1038/s41597-025-06212-0

**Published:** 2025-10-30

**Authors:** S. Turner, J. Hannaford, L. J. Barker, G. Suman, A. Killeen, R. Armitage, W. Chan, H. Davies, A. Griffin, A. Kumar, H. Dixon, M. T. D. Albuquerque, N. Almeida Ribeiro, C. Alvarez-Garreton, E. Amoussou, B. Arheimer, Y. Asano, T. Berezowski, A. Bodian, H. Boutaghane, R. Capell, H. Dakhlaoui, J. Daňhelka, H. X. Do, C. Ekkawatpanit, E. M. El Khalki, A. K. Fleig, R. Fonseca, J. D. Giraldo-Osorio, A. B. T. Goula, M. Hanel, S. Horton, C. Kan, D. G. Kingston, G. Laaha, R. Laugesen, W. Lopes, S. Mager, M. Rachdane, Y. Markonis, L. Medeiro, G. Midgley, C. Murphy, P. O’Connor, A. I. Pedersen, H. T. Pham, M. Piniewski, B. Renard, M. E. Saidi, P. Schmocker- Fackel, K. Stahl, M. Thyer, M. Toucher, Y. Tramblay, J. Uusikivi, N. Venegas- Cordero, S. Visessri, A. Watson, S. Westra, P. H. Whitfield

**Affiliations:** 1 UK Centre for Ecology & Hydrology, Wallingford, United Kingdom; 2 Irish Climate Analysis and Research Units (ICARUS), Dept. Of Geography, Maynooth University, Maynooth, Ireland; 3 Polytechnic Institute of Castelo Branco, Castelo Branco, Portugal; 4 Institute of Earth Science (IES), University of Évora, Évora, Portugal; 5 Centre for Climate and Resilience Research (CR)2, University of Chile, Santiago, Chile; 6 University of Parakou, Parakou, Benin; 7 Swedish Meteorological and Hydrological Institute, Norrköping, Sweden; 8 University of Tokyo, Tokyo, Japan; 9 Gdansk University of Technology, Gdansk, Poland; 10 Gaston Berger University, Saint-Louis, Senegal; 11 University of Annaba, Annaba, Algeria; 12 LMHE, Ecole Nationale d’Ingénieurs de Tunis, Université Tunis El Manar, Tunis, Tunisia; 13 Ecole Nationale d’Architecture et d’Urbanisme, Université de Carthage, Carthage, Tunisia; 14 Czech Hydrometeorological Institute, Prague, Czechia; 15 Nong Lam University, Ho Chi Minh City, Vietnam; 16 King Mongkut’s University of Technology Thonburi, Bangkok, Thailand; 17 International Water Research Institute, Mohammed VI Polytechnic University, Ben Guerir, Morocco; 18 Norwegian Water Resources and Energy Directorate, Oslo, Norway; 19 Pontificia Universidad Javeriana, Bogotá, Colombia; 20 University of Abobo-Adjamé, Abidjan, Côte d’Ivoire; 21 Czech University of Life Sciences Prague, Prague, Czechia; 22 University of Canterbury, Christchurch, New Zealand; 23 Swiss Federal Office for the Environment, Ittigen, Switzerland; 24 University of Otago, Dunedin, New Zealand; 25 University of Natural Resources and Life Sciences, Vienna (BOKU), Vienna, Austria; 26 Bureau of Meteorology, Canberra, Australia; 27 National Water and Sanitation Agency (ANA), Brasilia, Brazil; 28 Cadi Ayyad University, Marrakech, Morocco; 29 Universidad Politécnica de Madrid, Madrid, Spain; 30 Stellenbosch University, Stellenbosch, South Africa; 31 Da Nang University of Science and Technology, Da Nang, Vietnam; 32 Warsaw University of Life Sciences, Warsaw, Poland; 33 INRAE, RECOVER, Aix Marseille University, Aix-En-Provence, France; 34 University of Freiburg, Freiburg, Germany; 35 University of Adelaide, Adelaide, Australia; 36 South African Environmental Observation Network, Pretoria, South Africa; 37 Research Institute for Development (IRD), Montpellier, France; 38 Finnish Environment Institute (SYKE), Helsinki, Finland; 39 Chulalongkorn University, Bangkok, Thailand; 40 University of Saskatchewan, Saskatoon, Saskatchewan Canada; 41 Present Address: Institute of Earth Science (IES), University of Évora, Évora, Portugal

Correction to: *Scientific Data* 10.1038/s41597-025-04907-y, published online 18 April 2025

In this article the author’s name H. Dakhlaoui was incorrectly written as H. Dakhaoui. The original article has been corrected.

